# Amino Acid Transport and Metabolism Regulate Early Embryo Development: Species Differences, Clinical Significance, and Evolutionary Implications

**DOI:** 10.3390/cells10113154

**Published:** 2021-11-13

**Authors:** Lon J. Van Winkle

**Affiliations:** Department of Medical Humanities, Rocky Vista University, 8401 S. Chambers Road, Parker, CO 80134, USA; lvanwinkle@rvu.edu or lvanwi@midwestern.edu

**Keywords:** amino acid transport, amino acid metabolism, embryo development, epigenetic modifications, offspring

## Abstract

In this review we discuss the beneficial effects of amino acid transport and metabolism on pre- and peri-implantation embryo development, and we consider how disturbances in these processes lead to undesirable health outcomes in adults. Proline, glutamine, glycine, and methionine transport each foster cleavage-stage development, whereas leucine uptake by blastocysts via transport system B^0,+^ promotes the development of trophoblast motility and the penetration of the uterine epithelium in mammalian species exhibiting invasive implantation. (Amino acid transport systems and transporters, such as B^0,+^, are often oddly named. The reader is urged to focus on the transporters’ functions, not their names.) B^0,+^ also accumulates leucine and other amino acids in oocytes of species with noninvasive implantation, thus helping them to produce proteins to support later development. This difference in the timing of the expression of system B^0,+^ is termed heterochrony—a process employed in evolution. Disturbances in leucine uptake via system B^0,+^ in blastocysts appear to alter the subsequent development of embryos, fetuses, and placentae, with undesirable consequences for offspring. These consequences may include greater adiposity, cardiovascular dysfunction, hypertension, neural abnormalities, and altered bone growth in adults. Similarly, alterations in amino acid transport and metabolism in pluripotent cells in the blastocyst inner cell mass likely lead to epigenetic DNA and histone modifications that produce unwanted transgenerational health outcomes. Such outcomes might be avoided if we learn more about the mechanisms of these effects.

## 1. Introduction

The variety of known functions of amino acid transport and metabolism supporting oocyte and early embryo development are quite remarkable. Amino acids and their metabolites resemble classical signaling molecules, such as hormones and growth factors, as well as the substances needed to regulate histone and DNA epigenetic modifications [1,2]. In addition, some amino acids serve as osmolytes to balance hyperosmotic conditions encountered in the reproductive tract beginning after ovulation [3,4]. Strikingly, many of these amino acid transporters, receptors, and possibly even transceptors normally function primarily in the central nervous system (CNS) of adults.

For example, a CNS glycine neurotransmitter transporter and a glycine-gated chloride channel may work together in vivo to support the cleavage stage development of mouse embryos and probably embryos of other species [3,5]. The full nature of this cooperation remains to be explored. Similarly, other amino acids promote preimplantation development in “a growth factor-like manner,” although the mechanisms underlying these phenomena are still being elucidated [2].

In blastocysts, redundant mechanisms exist to insure a continued development in species with invasive implantation [6,7,8,9]. Normally, amino acid transport system B^0,+^ must take up leucine to trigger mTOR1 signaling in the trophectoderm during a critical window of time [6], but other mechanisms step in should this one fail [6,7,8,9]. This signaling ensures the development of the trophoblast motility needed to invade the uterine epithelium. System B^0,+^ is also expressed in the oocytes, but not the blastocysts, of mammalian species with noninvasive implantation, and even in ovipara oocytes, probably to supply nutrients to these cells [10,11] but also possibly to serve signaling functions. (See below.) These differences in the timing of expression of the B^0,+^ transporter—that is, beginning or exclusively in the oocytes of some species, and during blastocyst formation in others—is termed heterochrony, a process employed in evolution [10].

In the inner cell masses (ICMs) of mammalian blastocysts, and probably the stem cells of other multicellular species including plants, amino acid transport and metabolism support and regulate the maintenance of pluripotency [12]. For example, threonine transport and metabolism in the embryonic stem (ES) of mice and their progenitor (ICM) cells selectively foster the trimethylation of lysine residue 4 in histone H3 (i.e., H3K4me3 formation). H3K4me3 specifically supports ES and ICM cell proliferation and pluripotency in mice, humans, and probably other species [1]. These and other fascinating amino acid actions on oocytes and pre- and peri-implantation embryos are considered in more detail in the following sections and summarized in Table 1.

## 2. Amino Acid Transport and Signaling: Proline-Preferring Systems and Systems N, Gly, and B^0,+^

### 2.1. Proline-Preferring Systems

In recent studies, Morris and associates showed not only that proline (Pro) fosters preimplantation embryo development, but also that this amino acid improves the subsequent development, when present during in vitro fertilization (IVF), of mouse oocytes [2,13]. When present during IVF, Pro increased the blastocyst formation and the number of cells in ICMs, probably owing to a reduction of both mitochondrial activity and the production of reactive oxygen species. However, the presence of Pro during IVF did not raise the number of trophectoderm cells to the level achieved in vivo [13], and that should be a goal for conditions in vitro. These authors provide evidence that Pro uptake by oocytes likely occurs via the Pro-preferring amino acid transport system PROT, a Na^+^-dependent transporter, and two Na^+^-independent transporters (PAT1 and PAT2). Nevertheless, the ability of excesses of other, often beneficial amino acids to block Pro effects reminds us that a balance of conditions may produce the most desirable outcomes of in vitro culture. The ability of high concentrations of betaine, glycine, and histidine to partially inhibit Pro transport, either competitively or noncompetitively, may account, in part, for their ability to block the positive effects of Pro during IVF [13].

Even more fascinating are the direct effects of Pro on embryos during preimplantation development. Especially when early embryos are cultured under the hyperosmotic conditions of the oviductal fluid [3], Pro fosters blastocyst formation when they are grown at low density (LD), but such is also the case even when the embryos develop at high density (HD) [2]. A stimulation of embryo development occurs after the 2-cell stage. At HD, paracrine signaling among embryos is likely pronounced, whereas this signaling is minimized at LD. Somewhat surprisingly, the HD culture of embryos partially counteracts the negative effects of hyperosmotic media [2], thus providing redundant mechanisms to foster development in such conditions in oviductal fluid in vivo. (See also the consideration of glycine transport below.)

Partially owing to this redundancy to resist hyperosmotic stress, Pro seems to act on preimplantation embryos in a growth-factor-like manner [2]. Moreover, Pro activates mTOR1, Akt, and ERK signaling likely through the action of its transporter(s) as transceptor(s), intracellular signaling owing to Pro or its metabolites, or a combination of these mechanisms. Excesses of glycine, betaine, and leucine seem to counteract the benefits of Pro on development in part by slowing Pro transport. Independently, excess glycine reduces the number of cells per blastocyst at least in an isosmotic medium (i.e., 270 mOsm/kg) [2], so glycine need not act only by inhibiting the action(s) of Pro. Hence, the beneficial effects of various amino acids on preimplantation embryo development likely occur independently of each other in some instances and through interactions with one another in other cases. The elucidation of all of these mechanisms will help us develop clinically useful culture media that minimize the health risks to offspring and future generations. (See below.)

### 2.2. System N

System N preferentially transports glutamine (Gln). However, the evidence of system N’s expression in cleavage-stage embryos is circumstantial. We proposed an increase in system N activity at the 4- to 8-cell stage of development owing to an increase in the level of Gln in embryos at that time [19]. Consistent with this interpretation, Gln fosters preimplantation development beginning after the 2-cell stage [2]. The importance of Gln to preimplantation development has been known for decades [26,27,28], although the mechanism(s) of its action are only now coming to light. While Gln likely functions as an intracellular osmolyte in early embryos under hyperosmotic conditions [4], all we know about other actions of Gln to support preimplantation development is that it does not act through mTOR1 signaling [2]. Beyond the scope of the current discussion, system N is upregulated in the inner cell mass of mouse blastocysts during diapause and is needed there to maintain a diapausing state [29,30]. (See also Section 4.2 below.)

Owing to the growth factor-like nature by which Gln acts on preimplantation embryos, it does not function only as an osmolyte to resist hyperosmolar environments [2,4]. As for Pro, Gln fosters blastocyst formation in hyperosmotic media when early embryos are cultured at LD, although, to a lesser extent, such is also the case even when the embryos develop at HD in vitro [2]. Both paracrine activity and intracellular osmolytes likely contribute to this resistance to hyperosmolarity. Future studies should reveal the elusive mechanisms by which Gln promotes preimplantation embryo development. Despite its possible toxicity, Gln, or a dipeptide form of it, likely needs to be included in the media used to culture early embryos in order to promote the development of the healthiest possible conceptuses and adults [31,32].

### 2.3. System Gly

We first reported that glycine (Gly) protects cleavage-stage embryos from the detrimental effects of the hyperosmotic oviductal fluid-like medium owing to its uptake as an osmolyte against its total chemical potential and concentration gradients via system Gly [14,15]. Since then, this selective transport of Gly has been studied extensively in early embryos to demonstrate its function as an osmolyte [3,4]. Nevertheless, Gly clearly has other actions, as described in the section on Pro transport above. Some of these effects of Gly occur owing to the expression of a glycine-gated chloride channel in preimplantation conceptuses [5], whereas other mechanisms of Gly action are likely yet to be determined (e.g., interactions between the chloride channel and system Gly, as in the brain). In vivo, these effects of Gly occur in the context of developmental changes in the oviductal fluid.

While somewhat controversial, it seems likely that the oviductal fluid becomes more hyperosmotic as development proceeds from the one-cell to the two-cell stages [3]. Using each of two separate reports, this change can be calculated to range from 18 to 59 mM [33,34], with a total osmolality of mouse oviductal fluid sometimes exceeding 350 mOsmol/kg [3]. Moreover, the increases in Na^+^ and Cl^−^ concentrations in the oviductal fluid [34] could contribute somewhat [14] to a greater Gly accumulation by early embryos as the osmolarity increases [4]. Thus, the physiological conditions in oviductal fluid of about one mM glycine [35] and the hyperosmolality of the fluid interact to foster early embryo development.

Redundant mechanisms exist, however, to resist the otherwise detrimental effects of the hypertonic oviductal fluid, since insulin-like growth factors [36] as well as other embryos nearby [2] foster preimplantation embryo development in hyperosmotic media. Nearby embryos promote development apparently via paracrine signaling [2], that may include the production of extracellular vesicles by the conceptuses [37]. All these mechanisms are likely present in vivo to promote the development of healthy embryos and subsequently adults. An open question remains as to whether early embryos, developing in a hypothetically more physiological hyperosmotic medium containing Gly, are healthier than those developing in a hypotonic medium. That is, do embryos and adults that result from IVF and other assisted reproduction efforts get healthier the more we mimic in vitro the normal physiological conditions in vivo?

### 2.4. System B^0,+^

Amino acid transport system B^0,+^ was unusual when first characterized in mouse blastocysts because it accepted both cationic and zwitterionic amino acids as good substrates [38]. Subsequently, however, this Na^+^-dependent system was found to strongly prefer branched chain amino acids, such as leucine (Leu), and benzenoid substrates like tryptophan (Trp) [8]. The system is pivotal in promoting the implantation and further development of mouse, rat, and human blastocysts, and probably blastocysts of other species exhibiting invasive implantation [6,7,8,9,19]. System B^0,+^ fosters this type of implantation in at least two ways [8].

First, system B^0,+^ takes up Leu during a critical time period required to cause mTOR1 signaling in blastocysts [6]. This signaling leads to the development of the trophoblast motility needed to penetrate the uterine epithelium. Meanwhile, the system becomes relatively inactive in blastocysts in utero, but it becomes active again when blastocysts are removed from the uterus [8].

The physiological reasons for these changes in system B^0,+^ activity are likely two-fold. A suppression of system B^0,+^ activity may occur in blastocysts owing to the inhibition of the system by extracellular histones in uterine secretions [9,19,39]. It remains to be determined whether these histones are free in uterine secretions or sequestered in extracellular vesicles produced by uterine epithelial or other cells, e.g., [37,40]. Regardless of their origin, such actions of the histones must somehow be neutralized at the time of implantation [9,19,39]. Following a reversal of this possible histone action and, thus, the reactivation of system B^0,+^ in blastocysts in implantation chambers, the system would help to remove Trp from the surrounding fluid. Trp removal from the chambers likely prevents the immunological rejection of blastocysts by suppressing T-cell proliferation during the initial trophoblast penetration of the uterine epithelium [41,42].

Clinically, imbalances of system B^0,+^-preferred substrates may lead to unwanted developmental consequences. For example, the consumption of an excess of the system B^0,+^‘s substrate, isoleucine (Ile), during the pre- and peri-implantation periods of development led mice to deliver pups that were 9% larger than controls on day 19 of pregnancy, but the pups born on day 20 were 9% smaller than controls [43]. Other effects of excess Ile consumption during early development were significantly different fetal and placental growth rates between days 15 and 18 of pregnancy.

Ile supplementation may have inhibited Leu and Trp uptake by blastocysts about 15 h prior to and during their implantation, respectively [39]. Alternatively, since Ile deficiency is an effective activator of trophoblast endocytosis and lysosome production [44], excess Ile consumption during preimplantation embryo development could conceivably reduce the trophectoderm endocytic uptake of uterine fluid proteins and their digestion in lysosome. A decreased lysosomal protein digestion could limit the supply of threonine and lysine to ICM cells [1,19,24]. (See Section 4 below.) Dietary supplementation with the branched chain amino acids, Ile, Leu, and valine, has become part of the solution to some health problems [43]. Hence, the use of such supplements should be cautious in species such as humans, that employ invasive implantation.

More broadly regarding mTOR1 signaling and the development of trophoblast motility, a maternal low protein diet (LPD) during preimplantation development to the blastocyst stage led to a 25% reduction in the Leu concentration in mouse uterine fluid [45]. In a model, the latter authors proposed that the decreased uterine fluid Leu concentration results in less mTOR1 signaling, greater compensatory trophoblast pinocytosis and lysosome biogenesis, and increased histotrophic nutrition beginning at the blastocyst stage [46,47]. Consequently, offspring grow faster, have greater adiposity, and develop cardiovascular dysfunction, hypertension, neural abnormalities, and altered bone growth. This model may apply to all mammalian species, including humans, with invasive trophoblast implantation [46,47].

But what might be the function(s) of system B^0,+^‘s expression in species with noninvasive or no placentation? System B^0,+^ is expressed in unfertilized pig oocytes but not blastocysts [11]. Since mTOR1 helps regulate the development of oocytes, cleavage-stage embryos, and blastocysts (e.g., [48]), it is conceivable that system B^0,+^ acts in porcine oocytes via the leucine activation of mTOR1, as well as serving to nourish these cells [10]. Similarly, system B^0,+^ likely helps Xenopus oocytes grow prior to their deposition in fresh water. After deposition, the expression of the system is turned off likely to help retain the amino acids in the oocytes [10]. On the other hand, the activation of system B^0,+^‘s expression upon fertilization of sea urchin eggs should help them accumulate nutrients from sea water [10], especially owing to the system’s low K_m_ values for the accumulation of some amino acids [8]. Such changes in the timing of system B^0,+^’s transporter gene expression during the early development of mouse, pig, Xenopus, and sea urchin oocytes and embryos is termed heterochrony, and heterochrony is an important process employed in the evolution of new species [10].

## 3. Amino Acid Transport and Signaling Sometimes Includes Metabolism: Systems L, b^0,+^, and b^+^ or y^+^

### 3.1. System L

System L is present in preimplantation mouse embryos at all stages of their development [17,19,36]. While this system transports branched chain and benzenoid amino acids well, it also likely takes up methionine (Met) [20]. Consequently, the removal of Met from KSOM culture medium causes fewer zygotes to develop into blastocysts [16]. Of the embryos that do develop to the morula and blastocyst stages, their content of histone H3K4me3 was significantly decreased, likely because they did not have enough Met to support one carbon (1C) metabolism and H3K4 methylation [1,16,19,35]. As we shall see, H3K4me3 is needed to maintain pluripotent and proliferating cells in morulae and blastocysts (Section 4.1 below).

### 3.2. System b^0,+^

We also discovered an unanticipated, Na^+^-independent transporter of both cationic and zwitterionic amino acids in mouse blastocysts [49]. While initially it seemed that both types of substrates were equally accepted for transport, arginine (Arg) was soon found to be the highly preferred substrate [17,19,36]. Importantly, this transporter helped to reveal redundant mechanisms to support the development of trophoblast motility in blastocysts [6,7,8].

Not only Leu, but also Arg, by itself, supports the development of trophoblast motility in blastocysts in vitro through the activation of mTOR1 signaling [6]. Arg is also a precursor to both nitric oxide (NO) and polyamines, and all these substances foster cell motility [7]. In fact, polyamines partially overcome the rapamycin inhibition of mTOR1 and the block to the development of trophoblast motility in mouse blastocysts [8]. Moreover, uterine secretions foster the development of trophoblast motility in mouse blastocysts in vitro, but only when the secretions are obtained at a specific time several hours prior to the time of blastocyst implantation [6]. Since amino acids are likely present continuously in these secretions, we speculate that macromolecular constituents, possibly contained in extracellular vesicles of uterine fluid (e.g., [37]) are responsible for promoting trophoblast motility. This redundancy likely explains why one or the other of system B^0,+^ or system b^0,+^ knockout results in only small, nonlethal effects [9,50]. For other nutritional purposes, several alternative systems for the transport of cationic and zwitterionic amino acids are present in early conceptuses (e.g., system L above for zwitterionic amino acid transport) [17,19,36].

### 3.3. System b^+^ or y^+^

In the case of Arg nutrition, at least two cationic amino acid transporters (CATs), CAT1 and CAT2, are expressed throughout the preimplantation development of mouse embryos [19]. In early porcine embryos, one of these transporters (CAT1) is upregulated during the development in vitro vs. the development in vivo [51]. While the latter transport has been attributed to that catalyzed by the well-known amino acid transport system y^+^ first identified in adult tissue [18,52,53,54], a careful characterization of cationic amino acid transport in fertilized mouse eggs, cleavage-stage conceptuses, and blastocysts distinguished this transport from that of system y^+^ and termed it b^+^ [18]. For example, the K_i_ (and K_m_) values for Arg and lysine (Lys) transport via system y^+^ are nearly identical, but these values differ by an order of magnitude or more for transport by one-cell embryos and blastocysts (Table 2).

Perhaps the Arg uptake by early embryos is so important that CATs, normally taking up Arg and Lys equally efficiently in adults, are adapted in early embryos to insure Arg is accumulated regardless of the activities of other Arg transporters, such as b^0,+^. Moreover, CATs’ preference for Arg over Lys transport in blastocysts is even more pronounced than in one-cell embryos (i.e., the K_m_ for Lys uptake is nearly 10-fold higher in blastocysts than in one-cell embryos, while the K_m_ for Arg remains unchanged, Table 2, *p* < 0.01) [18]. The latter development change in CAT transport activity could help to insure a preferential Arg uptake in blastocysts even when the high-affinity Arg-preferring system b^0,+^ transporter is knocked out [50]. Such Arg transport would likely foster the development of the trophoblast motility needed to penetrate the uterine epithelium [6,7,8].

Because of the importance of Arg to reproduction and embryo development [6,7,8,19,35], it has been used as a prenatal dietary supplement in numerous studies. A systematic review and meta-analysis of 47 animal studies including 12 human investigations revealed beneficial effects of dietary Arg supplementation on fetal/birth weight, but only in complicated pregnancies [55]. In another recent review [56], it was emphasized that dietary Arg supplementation for the first seven days of pregnancy in the rat increased litter sizes by 30% while maintaining normal birth weights [57]. Although this Arg effect might help avoid early embryo losses in a variety of species, including humans, one can also argue that the effect might not be beneficial. For example, the average liter size in isonitrogenous control animals in the cited study was 11.4 ± 0.4 pups, which is not significantly different from the normal litter size of 11.0 in Sprague Dawley rats [58]. Thus, it might also be concluded that Arg supplementation saves less healthy embryos otherwise destined to fail in early pregnancy. Unfortunately, offspring growth and other parameters, such as blood pressure and blood glucose levels, were apparently not followed into adulthood in this study [57], so it is not known whether 30% of the experimental pups were less healthy than control pups.

It has also been speculated that the other cationic amino acid, Lys, might be deficient in low-protein diets [24]. Both an increase and a decrease in maternal dietary lysine produced smaller fetuses/birth weights in rats, e.g., [59,60]. Since Lys is not taken up efficiently by systems in early mouse embryos (e.g., Table 2), other possible mechanism(s) for the effects of Lys-deficient diets on early embryo development are discussed in Section 4.2, below.

## 4. Regulation through Amino Acid Signaling and Metabolism but Apparently Not Involving/Requiring Amino Acid Transporters in the Apical Membrane of the Trophectoderm

### 4.1. Threonine and Serine in the Inner Cell Mass

Morphologically distinct cell types first appear in preimplantation mammalian blastocysts a few days after fertilization [1,19]. The trophectoderm surrounds the inner cell mass (ICM) and will initiate implantation one to a number of days later depending on the species. The ICM gives rise to all other mammalian tissues, and these cells are used to produce embryonic stem (ES) cells. Consequently, ES cells are used as models for the ICM [1,19].

Murine (mES) and probably bovine embryonic stem cells require Thr to stay pluripotent and proliferate [21,61]. Threonine dehydrogenase (TDH) regulates the conversion of Thr to Gly and acetyl CoA in these ES cells. To control Thr consumption, posttranscriptional and posttranslational effectors as well as gene transcription maintain TDH activity [62]. Glycine produced from Thr in these ES cells is crucial to sustain the specific epigenetic modifications needed to preserve their pluripotency [63,64].

While mES cells maintain an optimal intracellular Thr concentration through Thr uptake from the culture medium and metabolism via TDH, the blastocyst trophectoderm must supply Thr to the ICM. A direct contact between trophoblast and ICM cells might supply amino acids to these pluripotent cells, while ICM cells bordering the blastocoelic fluid inside the trophectoderm could take up amino acids from this fluid [65]. Moreover, the apical membrane of the trophectoderm expresses at least 12 amino acid transport system activities that could help provide amino acids to the ICM [19,36]. However, Thr is not received with a low K_m_ value by any of these systems [19,36]. Consequently, blastocysts must use a different process to supply Thr to their ICM cells.

In this regard, Thr accumulates in mouse blastocysts as they develop, while most other amino acids do not [1,7,19,66]. Even without the presence of amino acids in vitro, the Thr content of blastocysts increases as the development proceeds. Since Thr is an essential amino acid in animals, the trophectoderm probably generates Thr by hydrolyzing protein in vitro as well as in utero [19]. Trophoblast cells can readily carry out this process via pinocytosis and the subsequent digestion of the protein they take up [67].

Based on studies with mES cells, ICM cells then likely use two Na^+^-dependent and at least one Na^+^-independent transport systems to take up Thr. These transporters were identified, respectively, as the obligate exchange ASC amino acid transporters, ASCT1 and ASCT2, and a system L exchange amino acid transporter [20]. One or more of these Thr transporters may function for signaling as a transceptor (i.e., a transporter that initiates signaling) [22], as well as by supplying the intracellular Thr metabolized for the specific epigenetic modifications needed to maintain mES cell proliferation and pluripotency.

By promoting cMyc expression, Thr transport initiates mTOR1 signaling in mES cells. This signaling does not proceed, however, upon the disruption of the lipid rafts within which Thr transporter(s) reside [22]. Thr transport may need undamaged rafts, or transport might cause caveolae in rafts to initiate signaling as a transceptor. Thr transport is inhibited by its analogue, 3-hydroxynorvaline (3-HNV), which likely slows mES cell proliferation in this way [20], in addition to slowing Thr metabolism by blocking TDH activity [21]. (See below.) Likely in these same ways, 3-HNV blocks the formation of cavitated blastocysts by pre-compacted morulae [21].

Because Thr transporter(s) seem to function as transceptor(s) [22], we predicted correctly that 3-HNV would block TDH-deficient hES cell proliferation [23]. While it is conceivable that 3-HNV could inhibit the proliferation of hES cells by replacing Thr residues in proteins [68], 3-HNV does not slow the proliferation of other human and mouse cell lines [21], so cell growth is not blocked through 3-HNV incorporation into proteins. An excess Thr rescue of 3-HNV-inhibited hES proliferation supports the hypothesis that the ES cells of all mammalian species require Thr transport itself for signaling, in addition to the signaling provided in most species by Thr catabolism for epigenetic histone modifications [23].

#### 4.1.1. The Glycine Cleavage System (GCS) Is Also Needed to Maintain ES Cells

**Figure 1:**
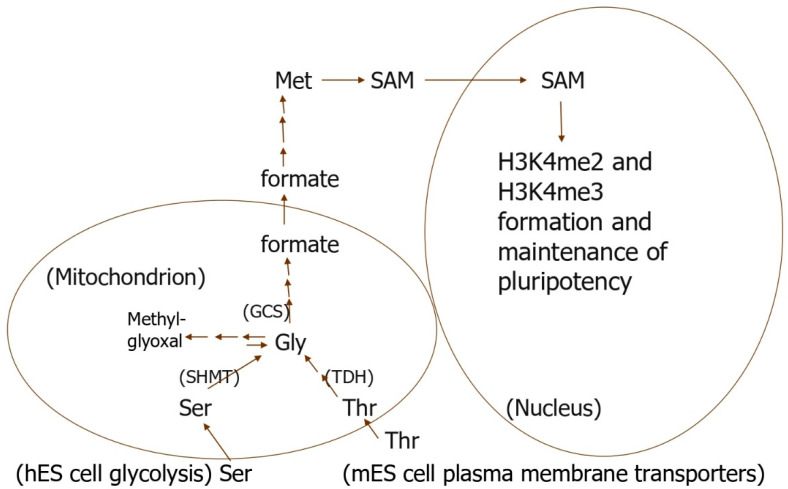
Mouse and probably most other mammalian ES cells require threonine (Thr) for the production of the 1C units needed to methylate histone H3K4. H3K4me3 formation is needed to maintain ES cell proliferation and pluripotency. Since other sources of 1C units cannot substitute Thr, we propose that a subpopulation of perinuclear mitochondria take up Thr, use it to form formate, and then selectively direct the formate to produce the S-adenosylmethionine (SAM) used in the nucleus to methylate H3K4. Since hES cells produce an inactive form of threonine dehydrogenase (TDH), they upregulate the expression of serine (Ser) synthesis enzymes probably to produce 1C units for H3K4me3 formation. GCS, glycine cleavage system; SHMT, Serine hydroxymethyltransferase. (The figure is a modification of those in reference [1]).

The GCS is needed to further process Gly generated by most mammalian ES and ICM cells owing to mitochondrial TDH activity (Figure 1). However, human ICM and ES cells produce inactive TDH, and they do not need it to generate Gly [69]. Instead, hES and probably their progenitor cells in the ICM greatly upregulate the expression of enzymes for serine (Ser) synthesis from glycolytic intermediates (Figure 1), whereas such upregulation does not occur in mES or mouse-induced pluripotent stem cells [64]. Gly is then likely generated from Ser by serine hydroxymethyltransferase (SHMT).

The further processing of Gly requires the glycine cleavage system (GCS) to maintain the pluripotency and proliferation of mES, hES, and likely other mammalian ES cells [64,70]. In this regard, the upregulation of GCS expression fosters the formation of induced murine and human pluripotent stem (iPS) cells. The GCS also prevents ES cell senescence by preventing methylglyoxal production (Figure 1) [64,70]. Instead, Gly is used in 1-carbon (1C) metabolism that fosters ES cell pluripotency and proliferation via specific epigenetic histone modifications [1,71,72,73]. This mitochondrial specialization is supported by glycolysis-generated ATP in ES cells [74]. Somewhat surprisingly, uncoupling protein 2 (UCP2) prevents pyruvate oxidation in mitochondria and, thus, shunts it to lactate, the final step in glycolysis [75,76].

#### 4.1.2. mES Cells Require Thr Catabolism for Specific Histone Modifications

Mouse ES cells stop proliferating when Thr is removed from the culture medium [21]. In the absence of Thr, the methylation of histone H3 to form H3K4me3 decreases dramatically, and this decrease is selective, since the methylation of DNA and other histone sites continues normally [63]. Somehow, mES cells take up Thr and direct it selectively to provide 1C units for H3K4 methylation. But how might such selection occur?

Thr catabolism in mES cells is performed by perinuclear mitochondria [77,78,79]. Thr may be selectively accumulated by a portion of these organelles specialized to do so (Figure 1). These specialized mitochondria might then produce formate from Thr and transport the formate to the cytosol, where it is converted to S-adenosyl methionine (SAM) methyl groups needed for nuclear H3K4 methylation [80,81]. The formate must somehow be directed specifically to H3K4 methylation sites, because the 1C units must originate from Thr (Figure 1) [63].

Another possibility is the selective direction of Thr by its plasma membrane transporter(s) to the portion of the mitochondria helping to form H3K4me3 in mES cells [1,20]. Nevertheless, the mitochondria would need to remain specialized for this purpose. Similarly, and despite being TDH-deficient, some hES cell mitochondria likely specialize in H3K4me3 formation via the GCS. H3K4me3 is required for hES as well as mES cells to proliferate and remain pluripotent [1,71,72,73].

#### 4.1.3. Why Doesn’t TDH Knockout Block Mouse Blastocyst Development?

Surprisingly, TDH knockout embryos appear to develop normally, and knockout adults are not sterile [82]. While both signaling via Thr membrane transport [22] and the subsequent Thr metabolism to form H3K4me3 [1,71,72,73] support mES cell pluripotency and proliferation, neither is sufficient alone to maintain such mES cell stemness [83]. Compensatory mechanisms to circumvent TDH knockout are, however, easy to envision, and may occur in mES progenitor cells in mouse blastocysts. For example, when Thr is deleted from the mES cell culture medium, the addition of excess Gly (plus pyruvate) to the medium maintains the H3K4me3 formation, which normally requires Thr [63]. Similarly, an increased supply of Gly occurs naturally inside TDH-deficient human iPS and hES cells when they upregulate serine synthesis to generate Gly and become/remain pluripotent (Figure 1) [64]. We propose that TDH knockout mES and their progenitor cells in blastocysts may adapt to mimic TDH-deficient hES cells (Figure 1).

A further understanding of this possible adaptation and regulation is likely essential for us to fully comprehend early embryo development in all mammalian species, since all species except humans express TDH, and even humans likely need specialized mitochondria in their ES and ICM cells. ES progenitor cells in the ICM give rise to all mammalian tissues and organs, so the clinical implications of environmentally altered epigenetic modifications seem to begin with such possible alterations in these cells. Much epigenetic reprogramming occurs during pre- and peri-implantation embryo development, e.g., [84]. Moreover, since Met is central to these epigenetic modifications (Figure 1) and to 1C metabolism in general, it might seem surprising, at first, to learn that a maternal low-protein diet (LPD) causes the Met concentration to increase in mouse uterine fluid on day 4 of pregnancy [45]. However, 1C metabolism might be sluggish owing to a LPD and to dependence on amino acids for 1C metabolism. Such slower 1C metabolism could result in a lower demand for Met in living cells including early embryos.

#### 4.1.4. Clinical Implications of Altered Epigenetic Histone and DNA Modifications

Maternal LPD consumption during early embryo development leads to several changes in trophectoderm behavior, including greater motility during the implantation in the uterus and an increased uptake of extracellular protein via endocytosis [46]. The hydrolysis of more protein releases more amino acids, including Thr, which might be delivered to ICM cells and could be taken up by at least three transporters [1,19,20]. A greater Thr transport into ICM cells may alter both signaling via its transceptor and H3K4me3 formation owing to changes in 1C metabolism. Also due to increased protein hydrolysis, trophoblast motility may increase owing to more Arg and Leu signaling and metabolism in these cells [6].

More efficient placentas also develop in association with a maternal LPD during early embryo development, possibly owing to a more robust trophoblast penetration of the uterine epithelium [46]. Similarly, modified epigenetic histone alterations in the primitive endoderm owing to a maternal LPD leads to the delivery of more nutrients to the embryo by the yolk sac placenta [46]. In addition, changes in dietary protein consumption during early embryo development cause ICM cell lineages to alter their epigenetic DNA modifications. These post-implantation as well as preimplantation alterations in the nutrients supplied to early embryos may affect the epigenetic modification of their DNA and histones, probably including H3K4.

#### 4.1.5. Future Generations Likely Experience Effects from DNA and Histones Modified during the Development of Their Ancestors

A maternal LPD, only during an F_0_ pregnancy, causes F_1_ rat offspring to produce an F_2_ generation with increased blood pressure and abnormal endothelial cells, likely due to transgenerational epigenetic histone and DNA changes [85]. Similarly, older maternal age or hampered placental function lead the F_0_ generation to transmit metabolic and cardiorenal changes to F_2_ rats, probably owing to epigenetic alterations [86,87]. In addition to the intracellular effects of these DNA and histone modifications, the histones might act extracellularly after their secretion into the uterine fluid by F_0_ and F_1_ females [9].

Furthermore, the genetic impairment of 1C folate metabolism in F_0_ mice suppresses epigenetic methylation, resulting in the altered development of wild-type mice over the following five generations [88]. In the latter case, DNA and histone modifications in F_1_ female germlines result in congenital malformations, while growth defects are caused by changes in the uterine environment. This uterine environment likely contains altered histones, because F_1_ wild-type females exhibited hypomethylation in their uterine cells. Histones in uterine fluid may affect blastocyst development and implantation by altering system B^0,+^ activity [9,39]. (See above.) The proposed histone hypomethylation may have contributed to both growth defects and congenital malformations because histones may have extracellular as well as intracellular effects.

Extracellular histones also provide one possible mechanism by which a paternal LPD adversely affects the health of their offspring [89]. Histones, modified owing to a LPD, might be present in the seminal fluid and dead sperm cells [90,91,92]. As for maternal epigenetic effects on future generations, paternal exposure to unhealthy environments adversely affects early embryo development through epigenetic transgenerational modifications [93,94].

### 4.2. Conversion of Lys to Glutamate in ICM Cells

While ES cells serve as models for ICM cells, the ES cell environment is not regulated in a physiologically normal way. In contrast, the immediate surroundings of ICM cells are controlled through interactions with the trophectoderm, e.g., [65]. Hence, data concerning the regulation of this environment need to be combined with findings of ES cell metabolism for a further understanding of the ICM cell function.

As for Thr, Lys is not a preferred substrate of any known transport system in the trophectoderm (e.g., Table 2) [19,36]. However, it may be needed in the ICM for glutamate (Glu) production [24]. We suggest that trophoblast cells take up protein by pinocytosis and hydrolyze the protein to generate Lys as well as Thr for the ICM [24]. The transport of Lys across the plasma membrane of ES and their progenitor cells in the ICM has not, to our knowledge, been characterized.

Nevertheless, hES cells remove Lys from their culture medium and release Glu to the medium [24], so the cells must express a transporter(s) to take up Lys. While Glu could conceivably be produced from Gln by extracellular glutaminase, we suggest that it is produced metabolically from Lys in hES cells and then released to the medium. This metabolism of Lys is possible because more Lys is consumed by hES cells than the amount of Glu they produce [24]. But what is the evidence that Lys is metabolized to Glu in hES cells, how does this metabolism matter to ICM cell pluripotency and proliferation, and what may be the clinical consequences of Lys deficiency owing to, say, LPDs?

#### 4.2.1. How Is Lys Converted to Glu in hES Cells?

Somewhat surprisingly, we found a relatively high expression of RNA encoding alpha-aminoadipic semialdehyde synthase (AASS) in hES cells [24]. AASS regulates the Glu synthesis from Lys in mouse and human brain, so such is likely the case for hES cells [95,96,97,98,99]. The resultant Glu is needed for normal brain and ES cell functioning through Glu signaling. Although the Glu-Gln cycle also produces Glu in the brain, it is insufficient for brain health [95,96,97,98,99]. Similarly, system N for Gln uptake is upregulated in mouse ICM cells to help maintain a diapausing blastocyst state [29,30], but diapause is characterized by a relatively slow cell division in blastocysts. Gln transport into ICM cells must decrease dramatically for the proliferation of ICM cells to continue [29,30], and glutaminase activity is likely relatively low in ES and their progenitor cells [24]. Thus, the conversion of Gln to Glu in ICM cells seems not to be a priority and is unlikely to contribute to the pool of Glu needed to support their pluripotency and proliferation. (See below.)

Hence, the pool of Glu produced from Lys seems to be used uniquely for signaling after the release from nerve and ICM cells [24,95,96,97,98,99]. Perhaps the Glu is extruded from mitochondria in the nerve and ICM cells and then preferentially released from the cells for autocrine and paracrine signaling. Contrariwise, instead of the proposed unique production of Glu from Lys, the trophoblast could conceivably supply Glu to ICM cells in a more direct manner. However, the Glu content of mouse blastocysts decreases as they develop [19,66], so there does not seem to be a concerted effort to supply free Glu directly to ICM cells as the development proceeds. So why is Glu needed outside of ES and ICM cells, and why might its presence outside ICM cells be carefully regulated?

#### 4.2.2. Function of Metabolically Produced Glu in ICM Cells

Mouse ES and, likely, ICM cells express mGlu5 metabotropic glutamate receptors, and they require the activation of these receptors to sustain self-renewal [100]. The mES cells produce Glu in vitro, and ICM cells likely do so in vivo, and such endogenous production of Glu fosters cell proliferation and the maintenance of pluripotency. The Glu activation of mGlu5 metabotropic glutamate receptors works to maintain a greater c-Myc expression in mES cells through the interaction with signaling by the leukemia inhibitory factor [101].

In this regard, we found hES cells to express mRNA encoding at least two such Glu receptors—metabotropic glutamate receptor 3 as well as 5 [24]. This mRNA is likely translated to produce glutamate receptor proteins in hES and ICM cells. We propose that endogenous Glu production from Lys in human as well as mouse ICM cells, and the release of this pool of Glu from the cells, help to maintain their pluripotency and proliferation.

#### 4.2.3. What Are the Possible Clinical Consequences of Lys Deficiencies Owing to LPDs?

When Lys is removed from the culture medium, hES and human iPS cells nearly stop proliferating [25]. Similarly, maternal LPDs may deprive ICM cells of Lys, because the concentration of Lys in mouse blastocysts is likely decreased by maternal LPD consumption [45]. Somewhat surprisingly, a maternal LPD increases the concentration of Met in the uterine fluid on day 4 of pregnancy in the mouse [45]. Such an increase in the Met concentration in vivo could lead to decreased blastocyst levels of the cationic amino acids-Arg and Lys—as also observed in mouse blastocysts in vitro in the presence of a physiological concentration of Met [102]. This decrease in the cationic amino acid concentrations in blastocysts occurs owing to the uptake of Met in exchange for (and exodus of) the cationic amino acids via system b^0,+^ [24].

The ability of Lys deprivation to nearly stop hES cell proliferation likely does not result only from the nutritionally essential nature of Lys. Human ES cells, grown without each of several other essential amino acids, continue to divide at almost normal rates [25]. Rather, without Lys, hES cells may not produce the specialized pool of Glu they need to bind metabotropic Glu receptors and, thus, remain pluripotent. (See above.)

Specialized pools of amino acids and their metabolites seem to be an especially important phenomenon in early embryos. For example, Thr is used selectively for H3K4me3 formation in mES and probably ICM cells in blastocysts (Figure 1). Other 1C sources cannot normally substitute Thr. Similarly, system B^0,+^ specifically directs Leu to sites of mTOR1 signaling in the blastocyst trophectoderm in order to foster the development of its motility [6]. Other transporters of Leu cannot substitute this B^0,+^ Leu transport, likely owing to the specialized intracellular Leu pool that B^0,+^ creates.

In contrast, mES cell proliferation does not appear to be as reliant on Glu production from Lys as hES cell proliferation [25]. The removal of Lys from the mES cell culture medium does not slow their proliferation [21], at least when the medium contains Glu [100,101]. However, for mammalian ES progenitor cells in the ICM, we suggest that Lys deprivation, owing to maternal LPDs, adversely alters the ICM production of tissues and organs in newborns and adults [24]. Unwanted effects of maternal LPD consumption during pre- and peri-implantation embryo development include metabolic syndrome and related disorders in adulthood [1,9,19,46,47].

## 5. Summary

Pro, Gln, and Gly each support preimplantation development apparently as growth factors, and their Na^+^-dependent transport against total chemical potential gradients allows them to serve as osmolytes to resist the otherwise detrimental effects of the hyperosmolar oviductal fluid. We still need to learn whether we can produce hyperosmotic conditions in vitro that allow oocyte fertilization and the development of the healthiest possible early embryos for transfer to the reproductive tract. Similarly, Leu and Arg transport into blastocysts via systems B^0,+^ and b^0,+^ both likely foster the invasion of the uterine epithelium. If the process is disturbed, however, owing to, say, a maternal LPD, the resultant embryos seem to give rise to adults with increased risk of having metabolic syndrome and related disorders. Finally, mouse and human ICM cells have different requirements for Thr and probably Lys uptake and metabolism [21,25]. Despite these differences, ICM cells of both species need the pertinent metabolic products, H3K4me3 and Glu, to remain pluripotent and proliferate. Disturbances in H3K4me3 and Glu production in ICM cells likely lead to transgenerational metabolic disorders.

## 6. Conclusions

### 6.1. Evolutionary Considerations

Amino acid transport system B^0,+^ not only supports the development of trophoblast motility in mammalian species with invasive blastocyst implantation, but it also seems to help nourish oocytes of species that do not penetrate the uterine epithelium after blastocyst attachment. Similarly, it may promote amino acid accumulation in Xenopus oocytes, prior to their deposition in fresh water, and in sea urchin eggs following fertilization. These changes in the timing of B^0,+^ expression are termed heterochrony—a well-known process employed in evolution [10].

Likewise, Thr transport and metabolism support stem cell maintenance in both animals and plants, so this reliance on Thr may be an ancient mechanism to conserve stem cell proliferation in multicellular organisms [12]. Similarly, Glu production from Lys seems to support the maintenance of pluripotency in ICM cells of mammalian blastocysts [24], while, in plants, this Glu production regulates their environmental responses and growth [103]. In the case of animal evolution, it seems likely that the Glu production from Lys was used first for signaling in ICM cells and, only later, for normal brain functioning after the evolution of this organ.

### 6.2. Several Ways to Foster the Development of Cleavage-Stage Embryos in the Hyperosmotic Oviductal Fluid

Cleavage-stage embryos likely develop in the hyperosmotic oviductal fluid [3]. While the osmolarities of oviductal and uterine secretions change as development proceeds, these secretions remain hyperosmotic at virtually all stages of preimplantation development [3,8]. Consequently, several mechanisms have evolved to help resist the potentially detrimental effects of these hyperosmotic conditions. The mechanisms include amino acid transport into the embryos as osmolytes, signaling by growth factors, such as insulin-like growth factors 1 and 2, and autocrine and paracrine effectors released by nearby embryos and cells of the reproductive tract [2,3,4,14,15,36]. Nevertheless, these changing hyperosmotic conditions in situ are likely beneficial to the reproductive process, although their possible advantages during embryo development are still to be determined. The elucidation of these benefits seems essential, however, in order to produce the heathiest possible life-long outcomes of assisted reproductive technology.

### 6.3. Redundant Mechanisms to Insure the Development of Trophoblast Motility and Implantation

Leu uptake by trophoblasts via system B^0,+^ fosters the mTOR1 signaling needed for the development of cell motility and the invasion of the uterine epithelium [6]. Nevertheless, alternate mechanisms, such as Arg transport by system b^0,+^, also serve this purpose [6,8]. Moreover, uterine secretions promote the development of trophoblast motility during a crucial interval several hours prior to implantation, and apparently not because the secretions contain amino acids. (See Section 2.4 above.) Consequently, neither system B^0,+^ nor system b^0,+^ knockout alone is detrimental to early embryo development or the fertility of the knockout mice [9,50]. Blastocyst implantation in the uterus is crucial to mammalian species’ survival, so it is not surprising that multiple mechanisms seem to fully support this process. Nevertheless, disturbances in the normal system B^0,+^ functioning, such as maternal consumption of a LPD, apparently lead to greater adiposity, cardiovascular dysfunction, hypertension, neural abnormalities, and altered bone growth in adults [46,47]. In this regard, a culture of mouse blastocysts for only a few hours without amino acids decreases mTOR1 signaling, which is only partially restored by amino acid addition [104].

### 6.4. Multiple Mechanisms to Maintain Pluripotent ICM Cells

Thr transporters in eukaryotic stem cells may function as transceptors to help maintain their proliferation [1,12]. Similarly, in most animals, TDH-regulated Thr metabolism, to form Gly and subsequently H3K4me3, likely maintains their pluripotent ES progenitor cells in the ICM [1,12]. In TDH-deficient hES progenitor cells, Gly and H3K4me3 are likely formed from Ser synthesized in the cells [64]. The proper maintenance of this H3K4me3 production through 1C metabolism is essential for the optimal development and production of healthy offspring. Gln metabolism can also contribute to ES cell maintenance through α-ketoglutarate production and epigenetic mechanisms [105], while Pro fosters ES cell differentiation [106].

In addition, the conversion of Lys to Glu may be needed to maintain pluripotent stem cells in the mammalian ICM [24]. The removal of Lys from the culture medium blocks hES (but not mES) cell proliferation almost completely [25], whereas the removal of Thr stops mES (but not hES) cell proliferation [21]. Hence, ES cells of these two species appear to have different needs for Lys and Thr uptake and metabolism. Nevertheless, the consumption of both Thr and Lys, and the production of Glu, by the bovine ICM [107], support the notion that most mammalian ICM cells require Thr and Lys to remain pluripotent. Moreover, the bovine trophectoderm produces both Thr and Lys [107], as we predicted in Section 4 above for the mouse and human trophectoderm. Disturbances of any of these processes in the ICM may lead to transgenerational epigenetic modifications with serious health consequences in adults [1,24]. (See Section 4 above.)

## Figures and Tables

**Table 1:** Effect of amino acid transporters/transport systems on early embryo development.

Transporter/System	Preferred Amino Acids	Effect [References]	Mechanism
**Cleavage-stage embryos**			
Proline-preferring	Proline	Blastocyst formation [2,13]	mTOR1, Akt, ERK signaling
System N	Glutamine	Blastocyst formation [2,4]	Growth factor-like; Osmolyte
System Gly	Glycine	Blastocyst formation [3,4,14,15]	Osmolyte in hypertonic oviductal fluid
System B^0,+^	Branched chain/Benzenoid	Oocyte nutrition in ungulates (e.g., pig) [11]	Amino acid uptake
System L	Bulky side chain	Blastocyst formation [16]	Methionine uptake
System b^0,+^	Arginine	Embryo nutrition [17]	Amino acid uptake/exchange
System b^+^	Arginine	Embryo nutrition [18]	Arginine uptake
**Blastocysts**			
System B^0,+^	Branched chain/Benzenoid	Development of trophoblast motility; Suppression of invading blastocyst rejection [6,7,8]	Leucine uptake initiates mTOR1 signaling; Tryptophan removal suppresses T-cells
System b^0,+^	Arginine	Development of trophoblast motility [6,7,8]	mTOR1, nitric oxide, polyamine signaling
System b^+^	Arginine	Development of trophoblast motility [6,7,8]	mTOR1, nitric oxide, polyamine signaling
ASCT1/2	Threonine ^1^	ICM cell pluripotency [19,20,21,22,23]	Transceptor; ^2^ Formation of H3K4me3
Lysine-preferring	Lysine ^1^	ICM cell proliferation [24,25]	Glutamate formation

^1^ Selectivity to be determined. ^2^ Transporter signaling.

**Table 2:** The K_i_/K_m_ values for Arginine and Lysine transport by cationic amino acid transporters (CATs) are nearly identical in adult tissues or during heterologous expression in Xenopus oocytes, but the values are one or two orders of magnitude different for CATs expressed in preimplantation mouse embryos (**, *p* < 0.01).

CAT Expression in:	K_i_ (K_m_) Values (Mean +/− SEM, mM) ^1^
	Arginine Lysine
Fibroblasts (y^+^)	0.041 ± 0.002 n.s. 0.040 ± 0.004
Hepatoma cells (y^+^)	0.20 ± 0.04 n.s. 0.14 ± 0.01
Xenopus oocytes (CAT2)	0.19 ± 0.03 n.s. 0.20 ± 0.03
One-cell embryos (b^+^_1_)	0.13 ± 0.04 ** 1.25 ± 0.18
Blastocysts (b^+^_2_)	0.084 ± 0.021 ** 8.10 ± 1.00

^1^ Data from [18,52,53,54], ** (*p* < 0.01), n.s. (not significant)

## Data Availability

All data are included in the manuscript.
